# Supplementary material for: Modeling glioblastoma heterogeneity as a dynamic network of cell states
Source: Mol Syst Biol. 2021 Sep 16;17(9):e10105. doi: 10.15252/msb.202010105 (PMC8444284; doi:10.15252/msb.202010105)
Supplement: Supplementary file 6 — Source Data for Figure 5 [file MSB-17-e10105-s004.zip › Figure5A_sourcedata/GSEA_3071/hallmarks_stateII.GseaPreranked.1623742536692/HALLMARK_PEROXISOME.html]

Details for gene set HALLMARK\_PEROXISOME[GSEA]

|  || Dataset | state23071 |
| Phenotype | NoPhenotypeAvailable |
| Upregulated in class | na\_pos |
| GeneSet | HALLMARK\_PEROXISOME |
| Enrichment Score (ES) | 0.26011133 |
| Normalized Enrichment Score (NES) | 1.1778706 |
| Nominal p-value | 0.2572707 |
| FDR q-value | 0.3234702 |
| FWER p-Value | 0.994 |
Table: GSEA Results Summary

  

Fig 1: Enrichment plot: HALLMARK\_PEROXISOME      
 Profile of the Running ES Score & Positions of GeneSet Members on the Rank Ordered List

  

| PROBE | GENE SYMBOL | GENE\_TITLE | RANK IN GENE LIST | RANK METRIC SCORE | RUNNING ES | CORE ENRICHMENT || 1 | MSH2 |  |  | 66 | 1.163 | 0.0528 | Yes |
| 2 | TOP2A |  |  | 72 | 1.132 | 0.1188 | Yes |
| 3 | IDH2 |  |  | 78 | 1.117 | 0.1838 | Yes |
| 4 | CTPS1 |  |  | 162 | 0.934 | 0.2189 | Yes |
| 5 | YWHAH |  |  | 273 | 0.781 | 0.2382 | Yes |
| 6 | CLN6 |  |  | 431 | 0.663 | 0.2390 | Yes |
| 7 | FADS1 |  |  | 498 | 0.629 | 0.2601 | Yes |
| 8 | PABPC1 |  |  | 1075 | 0.422 | 0.1436 | No |
| 9 | DHCR24 |  |  | 1253 | 0.386 | 0.1230 | No |
| 10 | FDPS |  |  | 1348 | 0.367 | 0.1217 | No |
| 11 | ITGB1BP1 |  |  | 1382 | 0.360 | 0.1349 | No |
| 12 | ECI2 |  |  | 1516 | 0.338 | 0.1223 | No |
| 13 | HRAS |  |  | 1538 | 0.334 | 0.1369 | No |
| 14 | SLC25A19 |  |  | 1542 | 0.333 | 0.1559 | No |
| 15 | CRABP2 |  |  | 1724 | 0.307 | 0.1297 | No |
| 16 | ISOC1 |  |  | 1825 | 0.296 | 0.1226 | No |
| 17 | ACSL4 |  |  | 2006 | 0.272 | 0.0945 | No |
| 18 | CAT |  |  | 2344 | -0.264 | 0.0273 | No |
| 19 | PRDX5 |  |  | 2373 | -0.267 | 0.0363 | No |
| 20 | GSTK1 |  |  | 2440 | -0.276 | 0.0365 | No |
| 21 | PEX2 |  |  | 2792 | -0.334 | -0.0300 | No |
| 22 | SMARCC1 |  |  | 2837 | -0.342 | -0.0205 | No |
| 23 | ECH1 |  |  | 2885 | -0.353 | -0.0112 | No |
| 24 | FIS1 |  |  | 3004 | -0.374 | -0.0180 | No |
| 25 | ACOX1 |  |  | 3121 | -0.404 | -0.0226 | No |
| 26 | ACAA1 |  |  | 3203 | -0.429 | -0.0170 | No |
| 27 | MVP |  |  | 3208 | -0.431 | 0.0076 | No |
| 28 | SLC25A4 |  |  | 3507 | -0.544 | -0.0334 | No |
| 29 | ATXN1 |  |  | 3586 | -0.598 | -0.0171 | No |
| 30 | IDH1 |  |  | 3842 | -0.832 | -0.0303 | No |
| 31 | CADM1 |  |  | 4042 | -1.568 | 0.0138 | No |
Table: GSEA details [plain text format]

  

Fig 2: HALLMARK\_PEROXISOME: Random ES distribution      
 Gene set null distribution of ES for **HALLMARK\_PEROXISOME**
